# Determinants of Breastfeeding Duration in Shiraz, Southwest Iran

**DOI:** 10.3390/ijerph17041192

**Published:** 2020-02-13

**Authors:** Mahnaz Zarshenas, Yun Zhao, Jane A. Scott, Colin W. Binns

**Affiliations:** 1 Fatemeh College of Nursing and Midwifery, Shiraz University of Medical Sciences, Shiraz 71348-14336, Iran; 2 School of Public Health, Curtin University, Perth 6845, Australia; Y.zhao@exchange.curtin.edu.au (Y.Z.); Jane.Scott@curtin.edu.au (J.A.S.)

**Keywords:** breastfeeding, prelacteal feeding, formula feeding, breastfeeding duration, Iran

## Abstract

A prospective cohort study was conducted in Shiraz in the south west of Iran to investigate breastfeeding from birth to six months of age. Mothers were recruited in a face-to-face interview within 48 h of giving birth in three public and two private hospitals (n = 700). They were then followed-up at 4, 12, 16, and 26 weeks postpartum in local Maternal and Child Health Clinics. Upon being discharge from hospital, 98.7% of mothers were breastfeeding and 74.3% were ‘fully’ breastfeeding, but only 29.9% of mothers had breastfed ‘exclusively’ since birth. The median duration of ‘full’ breastfeeding was 13 weeks and less than 1 week for exclusive breastfeeding. In a multivariable Cox proportional hazard regression, after adjustment, shorter durations of ‘exclusive’, ‘full’, and ‘any’ breastfeeding were associated with the introduction of a pacifier. The in-hospital use of formula and prelacteal feeds were also associated with a shorter duration of full and any breastfeeding. Breastfeeding on demand at 3 months and beyond was associated with a longer duration of breastfeeding. The risk factors associated with the premature discontinuation of breastfeeding identified in this study are all related to the “Ten steps to successful breastfeeding” and the Baby Friendly Hospital Initiative (BFHI). The principles that the BFHI provide are reaffirmed in this study as the basis for future breastfeeding promotion programs.

## 1. Introduction

Breastmilk provides the optimum nutrition for infants, protects against infection, promotes long-term health and is a crucial component of public health. Breastfeeding benefits are generally related to the amount of breastmilk given, measured by the duration of breastfeeding. Exclusively breastfed infants experience better health than those who are minimally breastfed or exclusively formula fed [1,2,3]. Infants who are not exclusively breastfed for the first months of their lives and are given other foods are more likely to be admitted to hospital with infections [4,5,6]. The World Health Organization (WHO), United Nations Children’s Fund (UNICEF), the American Academy of Pediatrics, and other international authorities recommend exclusive breastfeeding for the first six months, followed by the introduction of appropriate complementary foods. Breastfeeding should continue into the second year of life or longer [7,8]. The promotion of breastfeeding will result in important gains in health for Iran, which is a part of the Eastern Europe and Central Asia region discussed in the review of Rollins et al. [9]. 

In Iran, following the Islamic revolution, new targets and policies to improve breastfeeding were implemented and, beginning in 1980, the promotion and support of breastfeeding became a public health priority [10]. At this time, the National Committee for Breastfeeding Promotion was established, and training workshops, as well as other educational and research activities for the promotion of breastfeeding, were initiated across the country. These programs were targeted towards all health workers having contact with infants and mothers, including midwives, nurses, general physicians, pediatricians, and obstetricians. The Iranian government has supported the promotion of breastfeeding for several decades, and the rate of ‘any’ breastfeeding in 2006 was 90% and 57% at one and two years of age, respectively [10,11,12]. The exclusive breastfeeding rates 0 to 6 months, as measured by the cross-sectional Demographic Health Surveys were 44% in 2000 and 40% in 2004. [12,13]. In 2010 the exclusive breastfeeding up to 6 months of age was 53.1% in total and 27.8% and 62.8% in the urban and rural areas, respectively [12]. These rates are still below the national targets and international recommendations. 

The duration of breastfeeding is influenced by a complex mix of multiple factors, including sociodemographic, biomedical, and psycho-social factors [14,15,16]. These have not been investigated in detail in Iran in recent years. This is the first cohort study to investigate breastfeeding duration and associated factors from birth to six months in Iran. There have been no previous studies of exclusive breastfeeding and its determinants in Shiraz. The aim of this paper is to document factors associated with breastfeeding duration in Shiraz, a city located in the south west region of Iran.

## 2. Materials and Methods 

A prospective cohort study was conducted in Shiraz from June 2014 through to March 2015. In total, 700 women were recruited into the study by trained research assistants in face-to-face interviews within 48 h of giving birth in three public maternity hospitals and two private hospitals. Mothers were followed up at 4, 12, 16, and 26 weeks postpartum in local Maternal and Child Health Clinics when they attended for postpartum and infant care. At these times, the mothers were interviewed by trained staff to collect information about breastfeeding practices and the reasons for changing their infant feeding method via face-to-face interviews.

Mothers who were 18 years of age or older, with a healthy, full term (≥37 weeks) infant weighing 2500 g or more, were eligible to participate. Mothers who were not Iranian or living in Shiraz, too ill to answer or whose infant was admitted to the neonatal intensive care unit (NICU) for more than 72 h were ineligible to participate. Approval for the study was given by the Research Ethics Committee of the Shiraz University of Medical Sciences (209/2014) and the Curtin University Human Research Ethics Committee (HR 31/2014). All participants received verbal and written information about the study and were advised that their participation was voluntary and that they could withdraw at any time without prejudice. All women agreeing to participate in the study gave their signed informed consent.

### 2.1. Definitions of Breastfeeding

Breastfeeding terms used in the study were those recommended by WHO [17]. An infant was considered to be ‘exclusively’ breastfed if they had received breastmilk directly from their mother or expressed milk. Additionally, ‘exclusively’ breastfed infants must not have consumed any other liquids (except for mineral supplements, vitamin syrup, oral rehydration solution, and medications). To be ‘fully breastfed’, an infant received predominantly breastmilk as their source of nutrition with liquids including water, juice, ritual fluids, mineral supplements, and syrup, but no non-human milk. ‘Any breastfeeding’ applied if an infant was receiving breastmilk with or without non-human milk. Any breastfeeding includes all infants receiving breastmilk, including exclusive and full breastfeeding. The initiation of breastfeeding is defined as the infant being placed on the breast or receiving expressed breastmilk within 48 h of birth.

### 2.2. Main Outcome Variables

The three outcome variables investigated in this study were the risk of ceasing ‘exclusive’, ‘full’, and ‘any’ breastfeeding up to 26 weeks postpartum. In the baseline and follow-up questionnaire, mothers were asked their current feeding method and, if relevant, the age of their infant, in weeks, when they had first received infant formula, other beverages (e.g., water, juice, animal milks), and complementary foods, or when they had stopped breastfeeding. These data were used to estimate the duration of ‘exclusive’, ‘full’, and ‘any’ breastfeeding.

### 2.3. Explanatory Variables

The explanatory variables examined as potential determinants were derived from the literature and included maternal and infant characteristics such as maternal age, the mother’s level of education, the mother’s employment status, infant weight, the use of a pacifier; and biomedical and hospital practices, including the method of delivery, parity, body mass index (BMI) before pregnancy, antenatal class attendance, time since birth of the first breastfeed, whether the mother was taught how to attach, whether the infant was given a prelacteal feed of formula while in hospital, and ‘feeding on demand’ at one, three, and four months [4,5,6] (Supplementary Materials Table S1).

### 2.4. Data Analysis

The IBM SPSS statistics for Windows 24.0 (IBM Corp, Armonk, NY, USA) was used to analyze the data. The Kaplan–Meier method was used to determine the prevalence of ‘exclusive’, ‘full’, and ‘any’ breastfeeding at 4, 12, 16, and 26 weeks postpartum. A bivariate Cox proportional hazard regression model was used initially to evaluate the association between each of the explanatory variables and the duration of ‘exclusive’ breastfeeding for mothers whose infant did not receive prelacteal feeds or formula in hospital, and ‘full’ and ‘any’ breastfeeding for all mothers. Factors found to be associated with breastfeeding duration at 20% (*p* ≤ 0.2) in the bivariate (unadjusted) analysis were included in a multivariable Cox proportional hazard regression model and adjusted for variables/potential confounders to explore the adjusted risk of stopping breastfeeding. The backward elimination method was applied to obtain a parsimonious model and only variables with a *p* value of less than 0.05 were retained in the final model. Crude (unadjusted) hazard ratios (CHR) or adjusted hazard ratios (AHR) together with their 95% confidence are reported. The Homosmer and Lemeshow goodness-of-fit test was performed to assess the model performance of each Cox proportional hazard model using Stata 14 [18,19,20].

## 3. Results

In total, 1572 mothers were screened for the study, 711 met the inclusion criteria, and 700 (97.4%) of those eligible, agreed to participate. Almost all of the ineligible mothers lived outside the catchment area. Of these women, 672 (96%) attended the 4 weeks follow-up visit, 662 (94.6%) attended both the 12 and 16 weeks visits, and 660 (94.3%) attended the 26 weeks follow-up visit. The majority of recruited mothers were younger than 30 years of age (59.3%), educated to high school or university level (80.3%), and were primiparous (54.6%). A high percentage (70.8%) were delivered by caesarean section (Supplementary Table S1).

Upon being discharged from hospital, 98.6% of mothers were still breastfeeding (‘any’) and 74.3% were ‘fully’ breastfeeding. Only 29.9% of mothers had breastfed their infants exclusively since birth. By 26 weeks of age, 87% of infants were still receiving breastmilk, 28% of infants were ‘fully’ breastfed, but only 1% of infants had been exclusively breastfed since birth (Table 1). The median duration of full breastfeeding was 13 weeks and exclusive breastfeeding was less than 1 week (0.7).

### 3.1. Reasons for Stopping Breastfeeding Before 6 Months

Mothers were asked for the main reason that they had stopped breastfeeding before 26 weeks. Of the 83 who had stopped breastfeeding, just under one third (n = 25) of these gave the reason that the baby was refusing to breastfeed and one fifth (n = 17) reported insufficient milk supply (Table 2).

### 3.2. Maternal and Infant Characteristics, Biomedical Factors, and Hospital Practices Associated with ‘Exclusive’, ‘Full’, and ‘Any’ Breastfeeding Duration

In the bivariate Cox regression analysis, compared to university educated women, those women who had not completed high school were less likely to have ceased ‘exclusive’, ‘full’ and ‘any’ breastfeeding before 26 weeks, as were those who were demand fed at 3 and 4 months postpartum. A number of hospital practices were associated with the duration of breastfeeding. For instance, women who had delivered by caesarean section, had not breastfed within the first hour of delivery or whose infant had received formula in hospital were more likely to have ceased ‘full’ and ‘any’ breastfeeding before 26 weeks. Women whose infants had used a pacifier were more likely to have ceased ‘exclusive’, ‘full’, and ‘any’ breastfeeding before 26 weeks. (Supplementary Materials, Table S1).

In the multivariable Cox regression, after the adjustment, mothers who introduced a pacifier were more likely to cease ‘exclusive’ (AHR 3.12, 95% CI (confidence interval) 1.10, 1.55), ‘full’ (AHR 1.80, 95% CI 1.48, 2.19), and ‘any’ (AHR 29.31, 95% CI 7.18, 119.60) breastfeeding compared with those who did not give their infant a pacifier. Prelacteal feeds were associated with the risk of early cessation of ‘full’ breastfeeding (AHR 1.31, 95% CI 1.07, 1.61), while the provision of formula in hospital was associated with the risk of early cessation of ‘full’ (AHR 3.15, 95% CI 2.59, 3.83) and ’any’ (AHR 1.65, 95% CI 1.08, 2.52) breastfeeding. Demand feeding to 3 months was associated with a reduced risk of early cessation of ‘exclusive’ (AHR 0.61, 95% CI 0.43, 0.86) and ‘any’ (AHR 0.42, 95% CI 0.25, 0.72) breastfeeding, while demand feeding to 4 months was associated with a reduced risk of early cessation of ‘full’ (AHR 0.66, 95% CI 0.55, 0.80) and ‘any’ (AHR 0.52, 95% CI 0.30, 0.93) breastfeeding (Table 3). All p values of the test for the three Cox proportional hazard models (Any BF: *p* = 0.3215; Full BF: *p* = 0.0957; Exclusive BF: *p* = 0.1571) are greater than 0.05 and indicated that the three models fitted the data adequately.

## 4. Discussion

Despite a near universal initiation rate of breastfeeding of 98.6%, less than one third of infants were breastfed exclusively while in hospital, declining to 15% at six months of age. These findings are significantly lower than those reported in other Iranian studies. For instance, Olang et al. in 2009 reported rates of 56% at four months and 27.7% at six months, while Mortazavi in 2015 reported a rate of 33% in the first month [10,21]. A mean duration of exclusive breastfeeding of 4.63 months was reported in a recent study conducted in the Fars province [22]. Part of the variation in reported rates may be due to a different methodology used in the studies. The accuracy and compatibility of the data for monitoring purposes depends on the use of standardized definitions of breastfeeding [23] and indicators of infant feeding practices recommended by WHO [24]. Data and information on exclusive breastfeeding since birth is rarely found in Iranian studies and exclusive breastfeeding is often reported using “current status” as an indicator. This evaluates the proportion of infants who have been exclusively breastfed in the past 24 h, whether the sample is uniformly distributed by age, and will report a period prevalence. It is likely that studies using this method misclassify infants as being ‘exclusively’ breastfed when they may have received prelacteal foods or infant formula in hospital, or formula following discharge, but not during the 24-h recall period. There is evidence that the use of the 24-h recall indicator recommended by WHO [24] over-reports the true prevalence of exclusive breastfeeding compared to recall since birth [25,26,27,28]. For example, a cross-sectional study conducted in Tehran using the 24 h recall method to determine the prevalence of exclusive breastfeeding reported a high exclusive breastfeeding rate of 46% at six months compared to the rate of 15% found in this study [29]. Studies are of very limited value if definitions are incorrect and if, in addition, the sample size is small [30].

Despite the substantial recognized benefits of breastfeeding to both infants and mothers, in the present study, one out of eight mothers had ceased breastfeeding by six months postpartum. Insufficient milk supply and infants refusing to suck were reported by mothers as the main reasons for stopping breastfeeding. An earlier study found that 39% mothers in Tehran, the capital city of Iran, cited inadequate milk supply as the main reason for discontinuing breastfeeding [31]. In a more recent study, Olang et al. reported that of the 5.3% of Iranian mothers who had stopped breastfeeding by six months postpartum, inadequate milk supply was the reason given by 28% of women [32]. Concern over an inadequate milk supply has been consistently documented as a significant contributor to the premature cessation of breastfeeding in Middle-Eastern [33,34,35] and Western studies [36,37]. While inadequate breastmilk is the most frequent reason given for the cessation of lactation, it has been estimated that less than 5% of mothers are unable to ‘fully’ lactate, due to anatomical and hormonal breast abnormalities [38].

The continuation of breastfeeding with the appropriate introduction of complementary foods until two years of age or beyond is recommended by WHO and UNICEF [8]. In the current study, 87% of mothers were still breastfeeding with 37% ‘full’ breastfeeding at 26 weeks. The median duration of ‘full breastfeeding’ was 13.1 weeks. The duration of breastfeeding amongst mothers varies between the different provinces of Iran. In North-Eastern Iran, at six months, 98% of infants were still predominantly breastfed [39], while in Tehran the rate of mixed breastfeeding was reported to be 23.4% at six months, with only 7.4% of infants still being breastfed at 22 months [29]. In contrast, a national study reported that 90% of mothers were still breastfeeding at 12 months [32]. These reports suggest that variations in breastfeeding duration may be partly related to socio-cultural factors. There are a number of ethnically diverse groups in Iran with long histories and different cultures and customs related to infant feeding. However, the duration of ‘full’ breastfeeding reported in the present study is notably higher than that reported for Kuwait, where only 2% of infants were ‘fully’ breastfed at six months, or in the UAE, where only 18.5% of infants were predominantly breastfed at four months, decreasing to 7.1% at six months [34,35]. The differences in duration of ‘full’ breastfeeding between Iran and other Middle-Eastern countries may be attributable to cultural differences and to concerted actions taken by the Iranian government during the last two decades to improve breastfeeding rates. In 1991, Iran was the first Middle-Eastern country to adopt the WHO International Code of Marketing of Breastmilk Substitutes, the National Committee of Breastfeeding Promotion was established in 1999, and in 2008, 80% of births occurred in Baby Friendly Initiative accredited hospitals [10,40].

The “Ten Steps to Successful Breastfeeding” were originally developed as a part of the Innocenti declaration and were endorsed by the World Health Assembly in 1992. Since that time, they have been the foundation of the WHO and UNICEF infant feeding policies, which includes the Baby Friendly Hospital Initiative (BFHI) [41]. This study provides further evidence of the importance of the “Ten steps to successful breastfeeding”, as each of the four risk factors associated with the premature discontinuation of one or more of ‘any’, ‘full’, and ‘exclusive’ breastfeeding were related to non-adherence to one of these steps [41]:

- Step 6: Do not provide breastfed newborns any food or fluids other than breast milk, unless medically indicated.
- Step 8: Support mothers to recognize and respond to their infants’ cues for feeding.
- Step 9: Counsel mothers on the use and risks of feeding bottles, teats, and pacifiers.

In this study, infant formula use was a strong predictor of the duration of ‘full’ and ‘any’ breastfeeding, and giving prelacteal feeds was a significant predictor of the duration of and ‘full’ breastfeeding. Other studies in Iran and Middle-Eastern countries have documented similar results. Examples include a retrospective survey in Iran that found that the provision of formula significantly increased the risk of early cessation of breastfeeding [32]. Another cohort study of 538 Iranian women also reported a negative association between the use of formula in hospital and exclusive breastfeeding in the first six months of life [29]. Similarly, a Turkish longitudinal study reported that formula feeding in hospital was associated with early weaning of exclusive breastfeeding [42].

While no other study has investigated the effect of prelacteal food on the duration of breastfeeding in Iran, a longitudinal study found that the introduction of fluids during the first month postpartum increased the risk of termination of predominant breastfeeding among Iranian women [39]. Prelacteal feeds and in-hospital formula supplementation has been reported to lead to nipple confusion and reduced milk supply [43]. It can also impact negatively on maternal confidence, and cause women to doubt the adequacy of their milk supply [44].

In the current study, feeding on demand at 4 months was a strong predictor of the duration of ‘full’ and ‘any’ breastfeeding. A cohort study conducted in Kuwait reported a significant association between demand feeding in hospital and the duration of ‘full’ breastfeeding [34]. Milk production is controlled by infant demand and the frequency of breastfeeding, and there is evidence that feeding on schedule may contribute to insufficient milk production [45].

Pacifier use was found to be a strong predictor of a shorter duration of ‘exclusive’, ‘full’, and ‘any’ breastfeeding. This finding is consistent with the results of a meta-analysis that found the use of a pacifier shortened the duration of ‘exclusive’ breastfeeding before six months and duration of ‘any’ breastfeeding before 24 months [46]. Olang and colleagues in a retrospective study, reported a significant association between offering a pacifier and early weaning among nursing mothers in Iran [32]. A study in Kuwait found a negative association between pacifier use before four weeks and duration of breastfeeding [34]. The timing of introducing a pacifier is crucial in mediating the outcomes of breastfeeding. Evidence indicates that the use of a pacifier in the first six weeks reduces the duration of ‘full’ breastfeeding [47] and reduces breastfeeding at six months [36]. The use of a pacifier is common practice in Western countries, for example, in Australia, eight out of ten mothers give their infant a pacifier [48]. Pacifier use encourages non-nutritive sucking and is associated with less frequent sucking at the breast, which reduces the production of milk [49]. Furthermore, effective breast stimulation requires a deep sucking action, while sucking on a pacifier is superficial, with a short, fast, and minimal effort [38]. This practice is discouraged by the WHO and UNICEF, and the avoidance of pacifier use is recommended as step nine of the “Ten steps of successful breastfeeding [50].

There are several limitations that should be considered when interpreting the results of this study. Recruitment was limited to Shiraz City, and while this is a typical Iranian city, it may not be possible to extrapolate the results to the whole of Iran. Further regional studies are required, and the methodology used in this study could be used as a basis for their design. The strength of this study is the high percentage of eligible mothers who agreed to participate in this study and the very high rate of follow-up. The prospective nature of the study, with frequent follow-up interviews and adherence to standard definitions of breastfeeding, add to the strength of the conclusions. One limitation of this study is that information on the underlying reasons for the use of formula and pacifiers and giving prelacteal foods were not included and require further detailed qualitative and quantitative studies.

## 5. Conclusions

Breastfeeding is important for the health of infants and their mothers. The duration of breastfeeding can be influenced by many variables, including biomedical factors, hospital practices, and social and environmental circumstances. Despite the widely accepted benefits of breastfeeding, the majority of mothers in Shiraz do not exclusively breastfeed their infants to six months. By the time the infant has been discharged from hospital, 70% have been given infant formula or other feeds, and this is associated with a shorter duration of breastfeeding. The risk factors associated with the premature discontinuation of breastfeeding identified in this study are related to the “Ten steps to successful breastfeeding” and the Baby Friendly Hospital Initiative. The principles of the BFHI are reaffirmed in this study as the basis for future breastfeeding promotion programs.

## Figures and Tables

**Table 1:** Prevalence (percentage and 95% CI) of breastfeeding in hospital to 26 weeks (n = 700).

Interview	Any BF	Fully BF	Exclusive BF
At discharge	98.6 (97.7–99.4)	74.3(71.1–77.5)	29.9 (26.5–33.3)
4 weeks	96.0 (94.5–97.4)	58.0 (54.3–61.7)	22.0 (18.8–25.1)
12 weeks	92.0 (89.9–94.07)	50.0 (46.2–53.8)	18.0 (15.07–20.9)
16 weeks	90.0 (87.7–92.3)	47.0 (43.2–50.8)	18.0 (15.07–20.9)
26 weeks	87.0 (84.4–89.57)	28.0 (24.6–31.4)	1.0 (0.24–1.7)

**Table 2:** Reasons for stopping breastfeeding.

Reason	*n*	%
Baby refused breastfeed	25	30.1
Maternal illness	20	24.1
Insufficient milk supply	17	20.5
Neonatal jaundice	11	13.3
Mastitis	10	12.0

**Table 3:** Maternal and infant characteristics, biomedical factors, and hospital practices independently associated with the risk of discontinuing exclusive ^1^, full, and any breastfeeding before 6 months.

Variables	Exclusive ^2^ Breastfeeding (*n* = 210)			Full ^3^ Breastfeeding (*n* = 700)			Any ^4^ Breastfeeding (*n* = 700)		
	AHR	95% CI	*p* Value	AHR	95% CI	*p* Value	AHR	95% CI	*p* Value
Mother’s age (years)									
<25	-				-		0.81		
25–29							0.57	0.47, 1.40	0.449
≥30							1.00	0.35, 0.93	0.024
Pacifier ever used									
Yes	3.12	1.10, 1.55		1.80			29.31		
No	1.00	2.13, 4.57	<0.001	1.00	1.48, 2.19	<0.001	1.00	7.18, 119.60	<0.001
Prelacteal feed given							-		
Yes	-			1.31					
No				1.00	1.07, 1.61	0.008			
Formula provided in hospital									
Yes	-			3.15			1.65		
No				1.00	2.59, 3.83	<0.001	1.00	1.08, 2.52	0.022
Demand fed at 3 months									
Yes	0.61	0.43, 0.86	0.005	NS			0.42		
No	1.00						1.00	0.25, 0.72	
Demand fed at 4 months									
Yes	NS			0.66			0.52		
No				1.00	0.55, 0.80	<0.001	1.00	0.30, 0.93	0.027

^1^ Includes only those women whose infants had not received prelacteal feeds and/or infant formula in hospital. ^2^ Adjusted for the mother’s education, birth weight, pacifier used, time until the first breastfeed, mother taught how to attach and position, infant demand fed at 3 months, infant demand fed at 4 months. ^3^ Adjusted for the mother’s education, the mother’s pre-pregnancy employment status, birth weight, pacifier used, delivery method, parity, the mother’s pre-pregnancy body mass index (BMI), time until the first breastfeed, mother taught how to position and attach, prelacteal feed given, formula given in hospital, infant demand fed at 3 months, infant demand fed at 4 months. ^4^ Adjusted for the mother’s age, the mother’s education, the mother’s pre-pregnancy employment status, birth weight, pacifier used, delivery method, parity, time until the first breastfeed, mother taught how to position and attach, formula given in hospital, infant demand fed at 1 month, infant demand fed at 3 months, infant demand fed at 4 months. AHR, adjusted hazards ratio; CI, confidence interval; NS, not significant in the adjusted model.

## Supplementary Materials

The following are available online at https://www.mdpi.com/1660-4601/17/4/1192/s1, Table S1: Participant characteristics and association of maternal and infant characteristics and the risk of cessation of exclusive, full and any breastfeeding before six months.
