# Supplementary material for: Determinants of Breastfeeding Duration in Shiraz, Southwest Iran
Source: Int J Environ Res Public Health. 2020 Feb 13;17(4):1192. doi: 10.3390/ijerph17041192 (PMC7068444; doi:10.3390/ijerph17041192)
Supplement: Supplementary file 1 [file ijerph-17-01192-s001.pdf]

International Journal of Environmental Research and Public Health

MDPI

Supplementary Materials: Determinants of Breastfeeding Duration in Shiraz, Southwest Iran

Table S1. Participant characteristics and association of maternal and infant characteristics and the risk of cessation of exclusive, full and any breastfeeding before six months.

			Exclusive[1] Breastfeeding			Full Breastfeeding			Any Breastfeeding		
**Variables**	***n***	**%**	**CHR**	**95% CI**	***p* value**	**CHR**	**95% CI**	***p* Value**	**CHR**	**95% CI**	***p* Value**
Maternal age											
<25	150	21.4	1.15	0.73, 1.82	0.550	0.85	0,67, 1.08	0.175	0.79	0.46, 1.35	0.391
25-29	265	37.9	0.83	0.56, 1.23	0.351	0.93	0.77, 1.14	0.504	0.61	0.38, 0.99	0.044
≥30	286	40.7	1.00			1.00			1.00		
Mother's education											
Primary to secondary	142	20.3	0.55	0.31. 0.98	0.043	0.66	0.51, 0.86	0.002	0.51	0.26, 0.99	0.049
High school	287	41.0	0.78	0.54, 1.13	0.190	0.91	0.75, 1.11	0.342	0.87	0.56, 1.35	0.539
University	271	38.7	1.00		1.00			1.00			
Mother's employment status											
Employed	125	17.9	1.00			1.00			1.00		
Unemployed	575	82.1	0.75	0.48, 1.17	0.207	0.80	0.64, 0.99	0.045	0.64	0.40, 1.02	0.060
Infant sex											
Male	353	50.4	1.00			1.00			1.00		
Female	374	49.6	0.87	0.61, 1.22	0.415	1.01	0.85, 1.21	0.914	0.94	0.62, 1.42	0.769
Infant weight birth (grams)											
2500-2999	224	32.0	1.00			1.00			1.00		
3000-3499	320	45.7	0.76	0.52, 1.13	0.176	0.83	0.68, 1.02	0.073	0.81	0.51, 1.27	0.355
≥3500	156	22.3	1.01	0.62, 1.65	0.972	1.03	0.81, 1.31	0.810	0.71	0.40, 1.27	0.247
Pacifier ever used											
Yes	379	56.4	3.05	2.10, 4.43	<0.001	2.14	1.77, 2.59	<0.001	38.25	9.41, 155.35	<0.001
No	293	43.6	1.00			1.00			1.00		
Method of delivery											
Vaginal delivery	209	29.9	1.00			1.00			1.00		
Caesarean delivery	491	70.1	1.07	0.74, 1.54	0.718	1.46	1.19, 1.78	<0.001	2.00	1.17, 3.44	0.012
Parity											
Primiparous	382	54.6	1.00			1.00			1.00		
Multiparous	318	45.4	0.80	0.56, 1.13	0.201	0.83	0.70, 0.99	0.048	0.55	0.35, 0.85	0.008
BMI pre-pregnancy (kg/m^2)											
<25	384	56.8	1.00			1.00			1.00		
≥25	292	43.2	1.16	0.82,1.65	0.398	1.18	0.98, 1.41	0.077	0.94	0.62, 1.44	0.788
Attended an antenatal class											
Yes	85	12.1	1.00			1.00			1.00		
No	615	87.9	0.99	0.60, 1.66	0.987	1.03	0.78, 1.35	0.855	1.72	0.80, 3.72	0.167

International Journal of
Environmental Research and Public Health

Variables	*n*	%	Exclusive[1] Breastfeeding CHR	95% CI	*p* value	Full Breastfeeding CHR	95% CI	*p* Value	Any Breastfeeding CHR	95% CI	*p* Value
Time to first breastfeed											
Less than one hour	225	32.1	1.00			1.00			1.00		
One hour or more	475	67.9	1.33	0.93, 1.91	0.120	1.53	1.26, 1.87	<0.001	1.94	1.16, 3.25	0.012
Taught how to position and attach											
Yes	513	73.3	1.00			1.00			1.00		
No or didn't need teaching	187	26.7	0.74	0.47, 1.17	0.199	0.82	0.67, 1.01	0.059	0.64	0.38, 1.08	0.093
Prelacteal feed given											
Yes	458	65.4	N/A			1.47	1.21, 1.78	<0.001	0.84	0.55, 1.28	0.420
No	242	34.5				1.00			1.00		
Formula provided in hospital											
Yes	244	34.9	N/A			3.54	2.93, 4.28	<0.001	1.91	1.27, 2.88	0.002
No	456	65.1				1.00			1.00		
Demand feed at 1 month											
Yes	486	72.3	0.84	0.58,1.22	0.361	0.99	0.81, 1.20	0.892	0.57	0.38, 0.88	0.010
No	186	27.7	1.00			1.00			1.00		
Demand feed at three month											
Yes	414	62.5	0.58	0.41, 0.82	0.002	0.64	0.53, 0.77	<0.001	0.24	0.15, 0.38	<0.001
No	248	37.5	1.00			1.00			1.00		
Demand feed at fourth											
Yes	379	57.3	0.52	0.37, 0.75	<0.001	0.60	0.50, 0.72	<0.001	0.21	0.13, 0.34	<0.001
No	283	42.7	1.00			1.00			1.00		

[1] Includes only those women whose infants had not received prelacteal feeds and/or infant formula in hospital. CHR crude hazards ratio, CI confidence interval, BMI Body Mass Index.
